# Association of bud and anther morphology with developmental stages of the male gametophyte of melon (Cucumis melo L.)

**DOI:** 10.18699/VJGB-22-18

**Published:** 2022-03

**Authors:** M.L. Nguyen, T.N.B.T. Huyen, D.M. Trinh, A.V. Voronina

**Affiliations:** Da Nang University – University of Education and Science, Da Nang City, Vietnam; Da Nang University – University of Education and Science, Da Nang City, Vietnam; Da Nang University – University of Education and Science, Da Nang City, Vietnam; Russian State Agrarian University – Moscow Timiryazev Agricultural Academy, Moscow, Russia

**Keywords:** male gametophyte, stages of microspore development, tetrad, pollen, flower bud, anther, Cucumis melo L, melon, мужской гаметофит, стадии развития микроспор, тетрада, пыльца, бутон, пыльник, Cucumis melo L., дыня

## Abstract

Correlations between the morphological features of f lower buds and the developmental stages of the male gametophyte are of great practical interest as a reliable marker that accelerates and simplif ies the selection of appropriate plant material for isolated microspore culture. Microspore culture enables one to quickly obtain many pure lines of different vegetable crops, but it has not yet been widely applied in the melon (Cucumis melo L.). To successfully apply this technique in a new culture, one has to optimize many of its elements: f irst, f ind the biological markers for selecting the f lower buds containing the microspores of certain development stages. The paper presents the results of research estimating the correlations between the length and diameter of the f lower buds, the length of the visual part of the corolla, the length of the anthers and the development stages of the male gametophyte in the F1 hybrid of the Kim Hong Ngoc melon. The strongest correlation (CC = 0.885) was found for the f lower bed diameter and a strong correlation (CC = 0.880), for the bud length. The corolla’s visual part was a less reliable morphological feature, and the anther’s length should not be used as a parameter to predict the developmental stages of the melon’s male gametophyte. It was also found that one anther could contain the microspores and pollen grains of different developmental stages. In the f lower buds less than 4 mm in length and 1.51 ± 0.02 mm in diameter prevailed tetrads, and in the buds 4.0–4.9 mm in length and 2.30 ± 0.02 mm in diameter, early microspores. The microspores of a middle stage of development prevailed in the f lower buds 5.0–5.9 mm in length and 2.32 ± 0.00 mm in diameter; mid and late vacuolated microspores, in the buds 6.0–8.9 mm in length and 2.96 ± 0.37 mm in diameter; and two-celled pollen, in the buds more than 9 mm in length and more than 3.97 ± 0.34 mm in diameter

## Abstract (ru)

Выявление корреляций между морфологическими признаками бутонов и стадиями развития мужского гаметофита представляет большой практический интерес, так как наличие надежного маркера ускоряет и упрощает отбор подходящего растительного материала для культуры изолированных микроспор. Культура изолированных микроспор позволяет в короткие сроки получать чистые линии многих овощных культур, однако для дыни (Cucumis melo L.) эта технология пока не получила распространения. Чтобы успешно применить данную технологию для новой культуры, необходимо оптимизировать множество ее элементов, прежде всего подобрать морфологические маркеры, позволяющие отбирать бутоны, которые содержат микроспоры определенных стадий развития. В нашей работе приведена оценка корреляции между длиной бутонов, диаметром бутонов, длиной видимой части венчика, длиной пыльников и стадиями развития мужского гаметофита дыни F1 гибрида Kim Hong Ngoc. Наиболее сильная корреляция установлена для диаметра бутонов, коэффициент корреляции составил 0.885. Сильная корреляция выявлена также для длины бутона, коэффициент корреляции 0.880. Длина видимой части венчика являлась менее надежным признаком, а длину пыльников не следует использовать в качестве параметра для прогнозирования стадий развития мужского гаметофита дыни. Отмечено, что в одном пыльнике одновременно находились микроспоры и пыльцевые зерна разных стадий развития. В бутонах длиной менее 4.00 мм и диаметром до 1.51 ± 0.02 мм преобладали тетрады; в бутонах длиной 4.0–4.9 мм и диаметром 2.30 ± 0.02 мм обнаружена наибольшая доля ранних микроспор, при этом преобладали микроспоры средней стадии развития; в бутонах длиной 5.0–5.9 мм и диаметром 2.32 ± 0.00 мм преобладали средние и поздние вакуолизированные микроспоры; в бутонах длиной 6.0–8.9 мм и диаметром 2.96 ± 0.37 мм – поздние вакуолизированные микроспоры; в бутонах длиной 9.0 мм и более, диаметром 3.97 ± 0.34 мм и более – двухклеточная пыльца.

## Introduction

The melon (Cucumis melo L.) is an economically important cultivated plant (Sebastian et al., 2010) grown in more than 1 mln ha of agricultural lands (FAOSTAT, 2019)1. For the time being, the most common melon has been F1 hybrids praised for their uniformity and high yield and providing proper biological protection of originator’s ownership.

Double haploids (DHs) are a valuable material of genetic research and selection, especially for F1 hybrids of agricultural plants (Shmykova et al., 2015b; Abdollahi et al., 2016). As of today, the technologies to obtain DHs have been developed for more than 250 species (Maluszynski et al., 2003) and many of them have been used to produce homozygous plants (Ferrie, Caswell, 2011).

Several publications describe successful melon DH production via pollination with irradiated pollen (Sauton, 1988; Hooghvorst et al., 2020) or via remote hybridization followed by embryo growing in vitro (Lotfi et al., 2003). There are also papers, whose authors cultivated the anthers (Abdollahi et al., 2016), unfertilized seedbuds (Shmykova et al., 2015а) and isolated microspores (Zhan et al., 2009; Chen et al., 2017) of members of the cucumber family.

The isolated microspore culture technique produces more regenerates compared to those of unfertilized seedbuds and anthers and is widely applied, especially in the cabbage family (Djatchouk et al., 2019; Kozar et al., 2020). Moreover, this technique excludes the somatic cells of a donor plant from the growing medium, leaving no doubt about the regenerates’ origin. However, it has never been applied to produce the DHs of members of the cucumber family.

DH production in isolated microspore culture can be affected by multiple factors such as microspore development stage; their genotype; growing medium composition; cell-rich fluid density; culture introduction technique; the effect of temperature and other cultivation conditions (Dunwell, 2010; Niazian, Shariatpanahi, 2020). The microspore development stage is the first factor to be accounted for when applying the isolated microspore culture technique to a new culture, because the development from tetrads to two-celled pollen may involve different stages (Touraev et al., 1991; Germanà, 2011). For example, to produce carrot DHs, it is recommended to cultivate tetrads and early microspores (Gorecka et al., 2010), while cultivation of middle and late microspores is most effective for callus induction in the balsam apple anther culture (Nguyen et al., 2019). And in the cabbage family, vacuolated microspores and two-celled pollen have the highest ability for embryogenesis (Telmer et al., 1992; Binarova et al., 1997; Custers et al., 2001; Babbar et al., 2004; Winarto, Teixeira da Silva, 2011)

Direct selection of separate microspores corresponding to a certain development stage to be cultivated in vitro seems to be an unresolvable problem. As a rule, plant material is selected based on such markers as the morphological characteristics of the flower buds and anthers (Takahata, Keller, 1991; Parra-Vega et al., 2013). In rape, soya, reddish, tomato, balsam apple, these markers include the length and widths of their flower buds (Weber et al., 2005; Han et al., 2014; Sumarmi et al., 2014; Adhikari, Kang, 2017; Nguyen et al., 2019). Several studies have proved that such parameters as the size and color of the flower cup as well as the cup/corolla length ratio and anther size can do the trick (De Moraes et al., 2008; Parra-Vega et al., 2013; Zhang et al., 2013). Since these parameters are species-specific, it is necessary to work out a specific protocol for the melon.

This paper presents the results of investigation into the morphological characteristics of the melon’s flower buds and anthers and the way they correlate with the plant’s microspore development stages

## Materials and methods

The flower buds of the F1 hybrid plants of the Kim Hong Ngoc melon produced by the Chia Tai Seed company (Thailand) were collected at 5:30–6:30 a. m. The buds of 3.6 to 15.6 mm in length (with 1-mm interval) were transported in ice and then stored for 24 hours at 4 °C. At least 10 buds were accounted for each of the intervals.

The buds’ morphological characteristics were assessed using a Zeiss Stemi 2000-C stereomicroscope (Suzhou Co., Ltd). Microspores were obtained from the anthers of each flower bud to be put on a glass slide into a drop of glycerin mixed with distilled water in proportion 1:1. Then the 15 μl of 2 % acetocarmine solution drop was added, covered with a cover slide and microscoped. For the purpose of fluorescent staining, the microspores extracted from the anthers were washed three times in PBS (8.0 g/l of NaCl, 0.20 g/l of KCl, 1.44 g/l of Na2HPO4 and 0.24 g/l of KH2PO4 were dissolved in the 3/4 of the required volume of distilled water; НCl and KОН were added to bring the pH value to 7.4, and distilled water was added to reach the finite volume), DAPI (4′,6-diamidino- 2-phenylindole) was added and then the microspores were studied using a Zeiss Axio Lab1 fluorescent microscope (Suzhou Сo., Ltd).

The microspore development stages were determined from the size and shape of the cells, the number of cell nuclei and their interposition (Vergne et al., 1987; Maluszynski et al., 2003; Blackmore et al., 2007; Zhang et al., 2013). In each specimen, the development stages of 100 microspores were observed. Such parameters as the presence of tetrads, early/ middle/late microspores and two-cell pollen were considered. The percentage of each development stage in a particular specimen was calculated as the ratio of the number of microspores related to a certain development stage to the total number of observed microspores multiplied by 100 %.

The statistical significance of the performed calculations was confirmed with ANOVA analysis and the Tukey test for α = 0.05. The correlation between the measured parameters and microspore development stages was determined using the linearity regression (R) and correlation (CC) coefficients. The collected data were described and processed with the R software

## Results and discussion

**Fig. 1.**
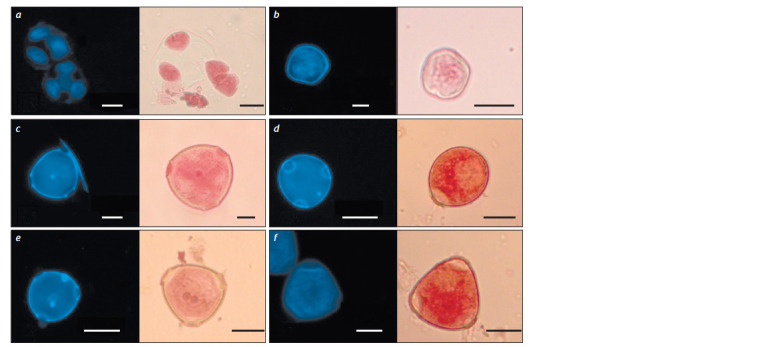
DAPI- and acetocarmine-dyed microspore development stages of Cucumis melo L.: a – tetrads, b – early microspores, c – mid microspores, d – late vacuolated microspores, e – early two-celled pollen, f – late two-celled pollen. Bar = 20 μm.

During the cytological analysis of melon flower buds, 6 stages of microspore development were observed. These included tetrads, early/middle/late vacuolated microspores, early/late two-celled pollen (Fig. 1).

**Fig. 2.**
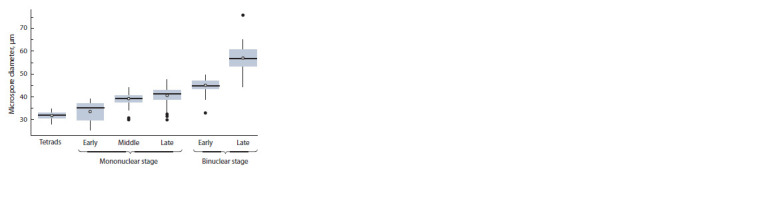
Changes in male gametophyte diameters related to their development stages.

The diameter of the microspores increased as they developed and reached their maximum at the stage of late twocelled pollen (Fig. 2). It has been noted that each stage was characterized by a certain shape and size of the cells. The diameter of the early microspores formed after tetrad degradation was 33.41 ± 4.34 μm; they were of uneven circular shape and had thin walls and large nuclei. The middle microspores were 39.06 ± 2.33 μm in diameter, had a round shape and a centered nucleus. The late microspores were round and had a wellexpressed three-lobed exine wall with the nucleus pressed to it by a big vacuole. Their diameter was 40.45 ± 3.26 μm. The cells of early two-celled pollen had 44.94 ± 2.65 μm in diameter with well-expressed two nuclei: a larger vegetative and a more vividly-colored generative one. The diameter of the late two-celled pollen comprised 56.93 ± 4.81 μm, its cell shapes varying from round to oval, so one anther could contain pollen grains of different shapes. The pollen’s cytoplasm became dense and nontransparent making it more difficult to observe the nucleus.

The results of the correlation analysis to bring together the morphological features of melon flower buds and corresponding microspore development changes enabled us to subdivide the buds into 6 groups. Each group could include microspores of different stages, so at least one of these stages prevailed (see the Table). It was noted that a single anther could have microspores that belonged to different development stages, which corresponds to the observations of other researchers who studied this issue in other cultures.

**Table 1.**
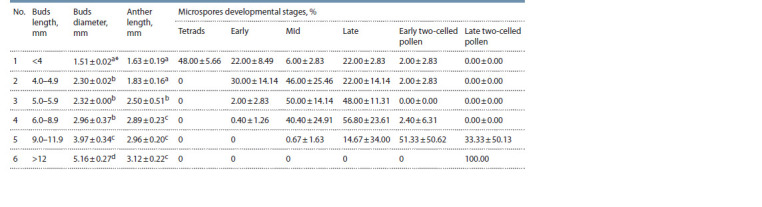
Correlations between flower-bud sizes, anther lengths and the stages of male gametophyte development in the melon Data marked with the same letters do not differ at р = 0.05.

**Fig. 3.**
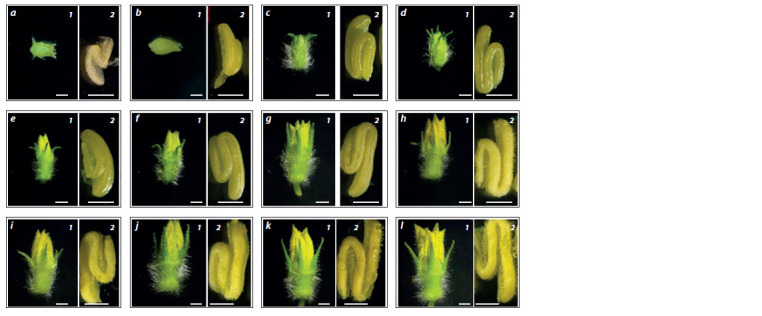
Changing the morphological characteristics of melon buds (1, bar = 20 μm) and anthers (2, bar = 10 μm) in relation to flower-bud sizes: a – 3.6–4.0 mm; b – 4.0–4.9 mm; c – 5.0–5.9 mm; d – 6.0–6.9 mm; e – 7.0–7.9 mm; f – 8.0–8.9 mm; g – 9.0–9.9 mm; h – 10.0–10.9 mm; i – 11.0–11.9 mm; j – 12.0– 12.9 mm; k – 13.0–13.9 mm; l – more than 14.0 mm.

The tetrads were found in green oval-shaped pubescent flower buds that were fully covered in sepals and had a length of less than 4 mm and a diameter of 1.85 mm (Fig. 3, a). The buds’ anthers were of light-beige color and had 1.6–1.63 mm in length.

The early microspores were found in flower buds of 3.8– 7.0 mm in length, their biggest portion (30 ± 14.14 %) concentrated in buds of 4.0–4.9 mm. The buds’ anthers changed their color to green-yellow, their length comprised 1.63–2.74 mm (see Fig. 3, b). The early microspores were found in smaller amounts compared to the other development stages.

The middle microspores concentrated in flower buds of 4.0 to 10.9 mm in length. The buds’ anthers were 2.15 ± 0.05 mm in length and had a yellowish glazing surface (see Fig. 3, c). The microspores prevailed (50 ± 14.14 %) in the buds of 5.0–5.9 mm in length. Such buds had a clear morphological difference from younger buds: their sepals were open, so one could see the corolla tip.

The late vacuolated microspores prevailed in buds of 6.0–8.9 mm in length. At this stage, the buds kept growing in size, so the corolla extended beyond the sepals. However, the anthers’ morphology remained unchanged (see Fig. 3, d–f ) as did their length

The early two-celled pollen prevailed in buds of 9.0– 12.0 mm in length (see Fig. 3, g–i). Their anthers’ length, compared to the previous stage, remained unchanged, their surfaces containing mature pollen grains.

As for buds larger than 12 mm in length, they contained only two-celled pollen. The transition from the late to mature stage was characterized by a small increase in bud size, its petals starting to open (see Fig. 3, j–l ). The anthers increased in size and opened too, so a large number of pollen grains could be seen on their surface.

Statistical analysis of anther lengths gave us linear regression coefficient R2 = 0.52, which meant that this parameter could not be used as a predictor of microspore development stages in the melon, which corresponded to the results obtained for some other cultures such as the tomato and aubergine (Segui-Simarro, Nuez, 2005; Salas et al., 2012). In (Adhikari, Kang, 2017), the authors obtained a similar coefficient (R2 = 0.59) when studying a relation between anther length and microspore development stages in the tomato

Many researchers recommend using flower-bud length to select proper plant material to cultivate isolated microspores for it is a convenient and reliable morphological parameter for many plant species. They also recommend bud diameter as an indicator for flower bud selection. A study published in 2019 demonstrated that the best results in the embryogenesis of lucerne microspores were obtained when cultivating late microspores from flower buds of 6.02–6.20 mm in length and 1.50–1.72 mm in diameter (Yi et al., 2019). In 2017, a correlation between flower-bud size (length and diameter), anther length and microspore development stages in the tomato was published (Adhikari, Kang, 2017).

**Fig. 4.**
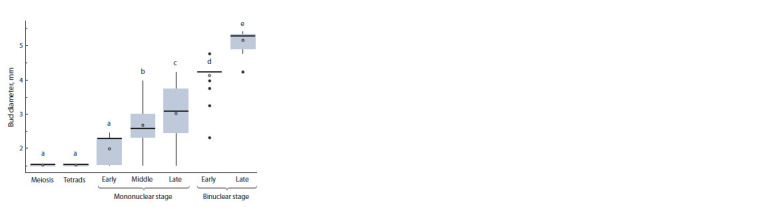
Correlation between the melon’s f lower bud diameter and male gametophyte development stages.

**Fig. 5.**
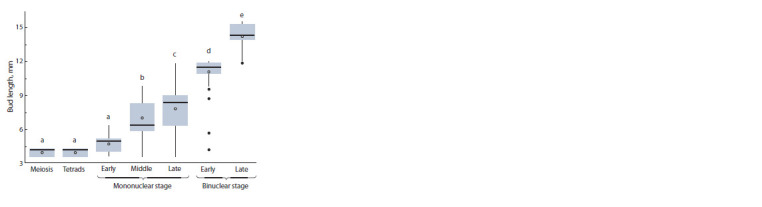
Correlation between the melon’s f lower bud length and male gametophyte development stages (a–e correspond to different bud groups).

In our study, a linear regression analysis showed there was a clear linear dependance ( p <0.05) between the flower-bud characteristics and microspore development stages. The regression coefficients (R2) varied from 0.767 to 0.783. The strongest correlation was for flower-bud diameter (r = 0.885, R2 = 0.783) (Fig. 4), followed by flower-bud (r = 0.880, R2 = 0.775) (Fig. 5) and anther (r = 0.876, R2 = 0.763) lengths, the last being the least reliable feature

## Conclusion

The correlation between the morphological characteristics of the flower buds and anthers of the melon (Cucumis melo L.) and the development stages of its microspores enables one to select a proper material for cultivation of isolated microspores in vitro. The characteristics in question are flower-bud diameter and length and the length of visible corolla. Since the correlation coefficient is higher for the diameter and length of flower buds, these parameters are easier to use.

The obtained results can be applied for further development of the technology to produce melon DHs in isolated microspore culture.

## Conflict of interest

The authors declare no conflict of interest.

## References

Abdollahi M.R., Najafi S., Sarikhani H., Moosavi S.S. Induction and
development of anther-derived gametic embryos in cucumber (Cucumis
sativus L.) by optimizing the macronutrient and agar concentrations
in culture medium. Turk. J. Biol. 2016;40:571-579. DOI
10.3906/biy-1502-55.

Adhikari P.B., Kang W.H. Association of floral bud and anther size
with microspore developmental stage in Campari tomato. Korean J.
Hortic. Sci. Technol. 2017;35(5):608-617. DOI 10.12972/kjhst.201
70065.

Babbar S.B., Agarwal P.K., Sahay S., Bhojwani S.S. Isolated microspore
culture of Brassica: an experimental tool for developmental.
Indian J. Biotechnol. 2004;3:185-202.

Binarovа P., Hause G., Cenklovа V., Cordewener J.H.G., Van Lookeren
Campagne M.M. A short severe heat shock is required to induce embryogenesis
in late bicellular pollen of Brassica napus L. Sex. Plant
Reprod. 1997;10:200-208. DOI 10.1007/s004970050088.

Blackmore S., Wortley A.H., Skvarla J.J., Rowley J.R. Pollen wall development
in flowering plants. New Phytol. 2007;174(3):483-498.
DOI 10.1111/j.1469-8137.2007.02060.x.

Chen J., Vanek E., Pieper M. Method for Producing Haploid, Dihaploid
and Doubled Haploid Plants by Isolated Microspore Culture. Patent
Int. Publ. No. WO 2017/017108 A1. Publ. date Feb. 2, 2017.

Custers J.B.M., Cordewener J.H.G., Fiers M.A., Maasen B.T.H., van
Lookeren Campagne M.M., Liu C.M. Androgenesis in Brassica:
a model system to study the initiation of plant embryogenesis.
In: Bhojwani S.S., Soh W.T. (Eds.) Current Trends in The Embryology
of Angiosperms. Dordrecht: Springer, 2001;451-469. DOI
10.1007/978-94-017-1203-3_18.

De Moraes A.P., Bered F., De Carvalho F.I.F., Kaltchuk-Santos E. Morphological
markers for microspore developmental stage in maize.
Braz. Arch. Biol. Technol. 2008;51(5):911-916. DOI 10.1590/S1516-
89132008000500006

Djatchouk T.I., Khomyakova O.V., Akinina V.N., Kibkalo I.A., Pominov
A.V. Microspore embryogenesis in vitro: the role of stresses.
Vavilovskii Zhurnal Genetiki i Selektsii = Vavilov Journal of Genetics
and Breeding. 2019;23(1):86-94. DOI 10.18699/VJ19.466. (in
Russian)

Dunwell J.M. Haploids in flowering plants: origins and exploitation.
Plant Biotechnol. J. 2010;8:377-424. DOI 10.1111/j.1467-7652.
2009.00498.x.

Ferrie A.M.R., Caswell K.L. Isolated microspore culture techniques
and recent progress for haploid and doubled haploid plant production.
Plant Cell Tissue Organ Cult. 2011;104:301-309. DOI 10.1007/
s11240-010-9800-y.

Germanà M.A. Anther culture for haploid and doubled haploid production.
Plant Cell Tissue Organ Cult. 2011;104:283-300. DOI 10.1007/
s11240-010-9852-z.

Gorecka K., Kowalska U., Krzyżanowska D., Kiszczak W. Obtaining
carrot (Daucus carota L.) plants in isolated microspore cultures.
J. Appl. Genet. 2010;51(2):141-147. DOI 10.1007/BF03195722

Han N., Kim S.U., Park H.Y., Na H. Microspore-derived embryo formation
and morphological changes during the isolated microspore
culture of radish (Raphanus sativus L.). Korean J. Hortic. Sci. Technol.
2014;32(3):382-389. DOI 10.7235/hort.2014.13170

Hooghvorst I., Torrico O., Hooghvorst S., Nogués S. In situ parthenogenetic
doubled haploid production in melon “Piel de Sapo” for
breeding purposes. Front. Plant Sci. 2020;11:378. DOI 10.3389/
fpls.2020.00378.

Kozar E.V., Domblides E.A., Soldatenko A.V. Factors affecting DH
plants in vitro production from microspores of European radish.
Vavilovskii Zhurnal Genetiki i Selektsii = Vavilov Journal of Genetics
and Breeding. 2020;24(1):31-39. DOI 10.18699/VJ20.592.

Lotfi M., Alan A.R., Henning M.J., Jahn M.M., Earle E.D. Production
of haploid and doubled haploid plants of melon (Cucumis melo L.)
for use in breeding for multiple virus resistance. Plant Cell Rep.
2003;21(11):1121-1128. DOI 10.1007/s00299-003-0636-3.

Maluszynski M., Kasha K., Forster B.P., Szarejko I. (Eds.) Doubled
Haploid Production in Crop Plants: A manual. Netherlands: Kluwer
Acad. Publ., 2003.

Nguyen M.L., Ta T.H.T., Huyen T.N.B.T., Voronina A.V. Anther-derived
callus formation in bitter melon (Momordica charantia L.) as
influenced by microspore development stage and medium composition.
Selskokhozyaystvennaya Biologiya = Agricultural Biology.
2019;54(1):140-148. DOI 10.15389/agrobiology.2019.1.140eng.

Niazian M., Shariatpanahi M.E. In vitro-based doubled haploid production:
recent improvements. Euphytica. 2020;216:69. DOI 10.1007/
s10681-020-02609-7.

Parra-Vega V., Renau-Morata B., Sifres A., Seguí-Simarro J.M. Stress
treatments and in vitro culture conditions influence microspore embryogenesis
and growth of callus from anther walls of sweet pepper
(Capsicum annuum L.). Plant Cell Tissue Organ Cult. 2013;
112(3):353-360. DOI 10.1007/s11240-012-0242-6.

Salas P., Rivas-Sendra A., Prohens J., Segui-Simarro J.M. Influence
of the stage for anther excision and heterostyly in embryogenesis
induction from eggplant anther cultures. Euphytica. 2012;184:235-
250. DOI 10.1007/s10681-011-0569-9.

Sauton A. Doubled haploid production in melon. In: Proceedings of the
EUCARPIA Meeting on Cucurbit Genetics and Breeding. Avignon–
Montfavet, France, 1988;06(01-02):119-128

Sebastian P., Schaefer H., Telford I.R.H., Renner S.S. Cucumber (Cucumis
sativus) and melon (C. melo) have numerous wild relatives
in Asia and Australia, and the sister species of melon is from Australia.
Proc. Natl. Acad. Sci. USA. 2010;107(32):14269-14273. DOI
10.1073/pnas.1005338107.

Segui-Simarro J.M., Nuez F. Meiotic metaphase I to telophase II as
the most responsive stage during microspore development for callus
induction in tomato (Solanum lycopersicum) anther cultures.
Acta Physiol. Plant. 2005;27:675-685. DOI 10.1007/s11738-005-
0071-x.

Shmykova N.A., Khimich G.A., Korotseva I.B., Domblides E.A. Prospective
of development of doubled haploid plants of Cucurbitaceae
family. Ovoshchi Rossii = Vegetable Crops of Russia. 2015a;3-4:
28-31. DOI 10.18619/2072-9146-2015-3-4-28-31. (in Russian)

Shmykova N.A., Shumilina D.V., Suprunova T.P. Doubled haploid
production in Brassica L. Vavilovskii Zhurnal Genetiki i Selektsii =
Vavilov Journal of Genetics and Breeding. 2015b;19(1):111-120.
DOI 10.18699/VJ15.014. (in Russian)

Sumarmi S., Daryono B.S., Rachmawati D., Indrianto A. Determination
of soybean (Glycine max L. [Merrill]) microspores development
stage based on the length of flower buds. J. Biol. Res. 2014;20:6-11.
DOI 10.23869/bphjbr.20.1.20142

Takahata Y., Keller W.A. High frequency embryogenesis and plant regeneration
in isolated microspore culture of Brassica oleracea L.
Plant Sci. 1991;74:235-242. DOI 10.1016/0168-9452(91)90051-9.

Telmer C.A., Simmonds D., Newcomb W. Determination of developmental
stage to obtain high frequencies of embryogenic microspores
in Brassica napus. Physiol. Plant. 1992;84:417-424. DOI 10.1111/
j.1399-3054.1992.tb04685.x.

Touraev A., Pfosser M., Vicente O., Heberle-Bors E. Stress as the major
signal controlling the developmental fate of tobacco microspores:
towards a unified model of induction of microspore/pollen embryogenesis.
Planta. 1996;200:144-152.

Vergne P., Delvallee I., Dumas C. Rapid assessment of microspore
and pollen development stage in wheat and maize using DAPI and
membrane permeabilization. Stain Technol. 1987;62:299-304. DOI
10.3109/10520298709108014.

Weber S., Unker W., Friedt W. Improved doubled haploid production
protocol for Brassica napus using microspore colchicine treatment
in vitro and ploidy determination by flow cytometry. Plant Breeding.
2005;124:511-513. DOI 10.1111/j.1439-0523.2005.01114.x.

Winarto B., Teixeira da Silva J.A. Microspore culture protocol for Indonesian
Brassica oleracea. Plant Cell Tissue Organ Cult. 2011;107:
305-315. DOI 10.1007/s112400110081z.

Yi D., Sun J., Su Y., Tong Z., Zhang T., Wang Z. Doubled haploid
production in alfalfa (Medicago sativa L.) through isolated microspore
culture. Sci. Rep. 2019;9:9458. DOI 10.1038/s41598-019-
45946-x.

Zhan Y., Chen J.F., Malik A.A. Embryoid induction and plant regeneration
of cucumber (Cucumis sativus L.) through microspore culture.
Acta Hortic. Sin. 2009;36(2):221-226.

Zhang C., Tsukuni T., Ikeda M., Sato M., Okada H., Ohashi Y., Matsuno
H., Yamamoto T., Wada M., Yoshikawa N., Matsumoto S.,
Li J., Mimida N., Watanabe M., Suzuki A., Komori S. Effects of the
microspore development stage and cold pre-treatment of flower buds
on embryo induction in apple (Malus×domestica Borkh.) anther
culture.
J. Jpn. Soc. Hortic. Sci. 2013;82(2):114-124. DOI 10.2503/
jjshs1.82.114.

